# Can Photobiomodulation Therapy (PBMT) Minimize Exercise-Induced Oxidative Stress? A Systematic Review and Meta-Analysis

**DOI:** 10.3390/antiox11091671

**Published:** 2022-08-27

**Authors:** Thiago De Marchi, João Vitor Ferlito, Marcos Vinicius Ferlito, Mirian Salvador, Ernesto Cesar Pinto Leal-Junior

**Affiliations:** 1 Laboratory of Phototherapy and Innovative Technologies in Health (LaPIT), Postgraduate Program in Rehabilitation Sciences, Universidade Nove de Julho (UNINOVE), São Paulo 03155-000, Brazil; 2 Postgraduate Program in Biotechnology, Oxidative Stress and Antioxidant Laboratory, University of Caxias do Sul, Caxias do Sul 95070-560, Brazil; 3 ELJ Consultancy, Scientific Consultants, São Paulo 01153-000, Brazil

**Keywords:** phototherapy, laser therapy, reactive species, exercise, oxidative damage

## Abstract

Oxidative stress induced by exercise has been a research field in constant growth, due to its relationship with the processes of fatigue, decreased production of muscle strength, and its ability to cause damage to the cell. In this context, photobiomodulation therapy (PBMT) has emerged as a resource capable of improving performance, while reducing muscle fatigue and muscle damage. To analyze the effects of PBMT about exercise-induced oxidative stress and compare with placebo therapy. Data Sources: Databases such as PubMed, EMBASE, CINAHL, CENTRAL, PeDro, and Virtual Health Library, which include Lilacs, Medline, and SciELO, were searched to find published studies. Study Selection: There was no year or language restriction; randomized clinical trials with healthy subjects that compared the application (before or after exercise) of PBMT to placebo therapy were included. Study Design: Systematic review with meta-analysis. Level of Evidence: 1. Data Extraction: Data on the characteristics of the volunteers, study design, intervention parameters, exercise protocol and oxidative stress biomarkers were extracted. The risk of bias and the certainty of the evidence were assessed using the PEDro scale and the GRADE system, respectively. Results: Eight studies (n = 140 participants) were eligible for this review, with moderate to excellent methodological quality. In particular, PBMT was able to reduce damage to lipids post exercise (SMD = −0.72, CI 95% −1.42 to −0.02, I^2^ = 77%, *p* = 0.04) and proteins (SMD = −0.41, CI 95% −0.65 to −0.16, I^2^ = 0%, *p* = 0.001) until 72 h and 96 h, respectively. In addition, it increased the activity of SOD enzymes (SMD = 0.54, CI 95% 0.07 to 1.02, I^2^ = 42%, *p* = 0.02) post exercise, 48 and 96 h after irradiation. However, PBMT did not increase CAT activity (MD = 0.18 CI 95% −0.56 to 0.91, I^2^ = 79%, *p* = 0.64) post exercise. We did not find any difference in TAC or GPx biomarkers. Conclusion: Low to moderate certainty evidence shows that PBMT is a resource that can reduce oxidative damage and increase enzymatic antioxidant activity post exercise. We found evidence to support that one session of PBMT can modulate the redox metabolism.

## 1. Background

In the current literature, it is already well evidenced that the production of reactive oxygen species (ROS) is associated with the development of pathogens such as cancer, cardiovascular diseases, diabetes mellitus, hypertension, and several neurodegenerative diseases [1,2,3,4]. The oxidation generated by ROS has the capacity to damage essential components of cells, causing oxidative stress, which decreases biological activity, leading to loss of energy metabolism, cell signaling, transport, and other important functions [1]. These harmful effects mentioned above occur due to the overproduction of ROS, which contributes to the phenomenon known as oxidative stress. In 2006, Helmut Sies and Dean Jones described the definition of oxidative stress as “an imbalance between oxidants and antioxidants in favor of oxidants, leading to an interruption of redox signaling and control, and/or molecular damage” [5,6]. On the other hand, the production of ROS at low or moderate levels has a beneficial effect on the organism, mainly because they act as intracellular signaling molecules for several physiological processes [5].

In recent decades, new studies have shown that physical exercise can induce oxidative stress [7,8,9,10]. The first study demonstrating these findings occurred in 1978 [11]. Dillard et al. concluded that lipid peroxidation occurs during exercise in subjects submitted to endurance exercise at 50% of VO2 max [11]. In later years, it was confirmed that the muscle contraction that occurs during exercise could generate ROS [7,12]. It is now known that the generation of ROS has an important influence on the contractile function of the muscle and the production of force. Consequently, ROS generation regulates the adaptive muscle responses provided by exercise and contributes to muscle fatigue [13,14]. In this context, there is evidence that higher levels of oxidative damage result in a more extended post exercise muscle recovery period [13,15]. In addition, excess ROS production during periods of intense training deserves attention from coaches and sports medicine professionals, as it can overcome the capacity of antioxidant defenses, impairing muscle performance and the quality of training sessions, which can result in overtraining and muscle injury [13,14]. Due to the impact of exercise-induced oxidative stress on sports performance, delayed onset muscle soreness and muscle injury biomarkers, there is a growing scientific interest in strategies/resources capable of reducing oxidative stress [15].

Recently, among the various resources researched to reduce the harmful effects of exercise, low-level laser therapy, also called photobiomodulation therapy (PBMT), has gained prominence [16]. The PBMT is defined as a form of light therapy that uses non-ionizing light sources, including lasers, light-emitting diodes and/or broadband light, in the visible (400–700 nm) and near-infrared (700–1100 nm) electromagnetic spectrum [17,18]. The first research with PBMT and oxidative stress was conducted in rats, and the results indicated an increase in antioxidant capacity [19], lower inflammatory [20,21] and oxidative damage markers levels [20,21].

Two years later, in 2008, the first randomized controlled trial (RCT) investigating the use of PBMT to improve athletic performance was published [22]. In the following years, there was an increasing amount of evidence supporting the use of PBMT to optimize performance and accelerate post exercise recovery. Two recent meta-analyses [23,24] demonstrated that PBMT significantly increased time and number of repetitions of exercises before onset of exhaustion, as well as reduced levels of muscle damage biomarkers in healthy adults, athletes, or physically active individuals. In addition, our group’s most recent review demonstrated that PBMT is more effective than cryotherapy in minimizing exercise-induced muscle damage [25]. However, none of these reviews investigated the effects of PBMT on oxidative damage and antioxidant defense.

Recently, a systematic review [26] sought to identify the PBMT effects on oxidative stress; however, the review focused on studies with animal models with induced muscle injury. The study based on eight trials with good methodological quality concludes that PBMT can be an effective short-term approach to reduce oxidative stress in muscle damage [26]. On the other hand, it is known that animal studies are often poor predictors of human reactions to exposure, due to the variety of metabolic pathways and metabolites of animals. This can lead to variations in efficacy and toxicity, in addition to divergent models of inducing diseases or injuries, with varying similarities to the human condition [27,28].

Thus, there is a need for a new systematic review of randomized trials with human subjects verifying the PBMT effects on exercise, specifically on its impact on oxidative damage and antioxidant defense biomarkers. Seeking to fill this gap in the literature, this systematic review aims to summarize the effects of PBMT on exercise-induced oxidative stress in healthy individuals.

## 2. Methods

The process of searching for and selecting articles met all the steps suggested by the Preferred Reporting Items for Systematic Review and Metanalysis Protocols–PRISMA-P [29]. The protocol review was prospectively registered in the International Prospective Register of Systematic Reviews (Registration Number: CRD42020206924).

### 2.1. Search Methods Used to Identify Studies

A literature review was developed without restrictions on the language of publication, from the earliest record up to September 2021 in electronic databases: The Cochrane Central Register of Controlled Trials (CENTRAL), Physiotherapy Evidence Database–PEDro, PubMed and Virtual Health Library (VHL) which include Lilacs, Medline and SciELO, Embase and Cumulative Index to Nursing and Allied Health Literature (CINAHL).

The main keywords DeCS/MeSH search terms used in the English language were: “oxidative stress”, “oxidative”, “stress”, “low-level light therapy”, “phototherapy”, “photobiomodulation”, “laser therapy” and “exercise”; for each concept, keywords were combined with the ‘AND/OR’ operator (see Supplementary Materials for full search strategy). This strategy was applied by two independent reviewers (JVF and MVF) who independently screened all titles and abstracts retrieved by the searches to identify potentially eligible trials. The last search took place on September 2021. We conducted an additional search on June, 2022 in order to identify potential studies published after September 2021.

The references of the eligible articles for this systematic review were analyzed to detect other potentially eligible studies. Authors were contacted by email for ongoing studies or when the confirmation of data or additional information was needed.

### 2.2. Eligibility Criteria

The inclusion criteria (see Box 1) for the study were articles that included research with humans in a randomized clinical trial as a study design. These articles were about the effects of PBMT on physical exercise, evaluating oxidative stress markers. Systematic reviews and meta-analyses, integrative reviews, case studies, observational studies, and trials that did not meet the purpose of the study or that were duplicated in the search databases were excluded. Trials that included subjects with neurological, orthopedic, or cardiovascular pathologies were also excluded.

Box 1 **Inclusion criteria.**

Design

  ● Randomized clinical trial

Participants

  ● Healthy subjects 18 years or older, who were enrolled in an exercise session or in a strength or aerobic training protocol

Intervention

  ● Photobiomodulation (PBMT) irradiation applied at any time (before or post exercise) of the physical exercise proposed

Comparisons

  ● Placebo therapy

Outcome measures

  ● Oxidative stress biomarkers (damage oxidative and antioxidant activi-ty)

### 2.3. Data Extraction

The data extracted from the eligible studies were about the characteristics of the participants (healthy adults), interventions (PBMT) compared to control and/or placebo groups, exercise protocol enrolled on (short- or long-term exercise, any type of exercise protocol), the moment of irradiation (before or after an exercise session), PBMT application parameters and assessment of oxidative stress using indirect biomarkers.

Data extraction was performed by a reviewer (JVF) and summarized through tabulation. All tabulated data were later reviewed by another reviewer (MVF). In the case of data that were not reported in the article, the corresponding author was contacted by email.

### 2.4. Data Syntheses and Analysis

The meta-analysis performed by one author (JVF) was reviewed by a statistician (TDM), using RevMan review management software (version 5.4, The Nordic Cochrane Centre, Copenhagen, Denmark) to summarize the treatment effect of PBMT on modulation of oxidative stress. Meta-analysis was only performed for those studies that compared PBMT to a placebo/sham group due to many comparisons. When a study had two or more groups submitted to PBMT but with different parameters or different application times (before or after exercise) compared to placebo, each group was considered as an independent study.

Pooled-effect estimates on continuous outcomes were conducted using post exercise values mean and standard deviations (SDs) in each of the time-points (post, 1 h, 24, 48, 72, and 96 h) for each group and were expressed as mean difference (MD) or standard mean difference (SMD). When the data values were not expressed in SD, SD values were calculated using methods recommended in the Cochrane Handbook for Systematic Reviews of Interventions [30]. As an example, SD was obtained from standard error (SE) of the mean multiplied by the root of the sample size.

Calculations were done using a random-effects model. A forest plot was created to present the MD or SMD with 95% confidence intervals (CIs). The heterogeneity between studies using the I^2^ statistic, with thresholds set as I^2^ = 25% (low), I^2^ = 50% (moderate), and I^2^ = 75% (high). The significance level was set a priori at *p* < 0.05.

### 2.5. Risk of Bias

The quality of the RCTs was classified by two researchers (JVF and MVF) using the PEDro scale, which is based on concealed allocation, intention-to-treat analysis, and adequacy of follow-up [31]. The Physiotherapy Evidence Database rating scale score is obtained by evaluating whether 11 criteria are fulfilled (score of 1) or not (score of 0). The first item does not contribute to the total score, and thus the final score is out of 10 [31,32]. PEDro scores of 0–4 are considered ‘poor’, 4–5 ‘fair’, 6–8 ‘good’, and 9–10 ‘excellent’, and for trials evaluating complex interventions (e.g., exercise), a total PEDro score of 8/10 is optimal [33].

### 2.6. Certainty of Evidence

The Grading of Recommendations, Development, and Evaluation (GRADE) system was used to evaluate the overall certainty of the evidence for each comparison, classifying the evidence into four levels of confidence, quality, or certainty: high, moderate, low, or very low [31,34]. According to this system, the assessment of the evidence set for an outcome depended on five factors that could reduce the confidence of these estimates: risk of bias (downgraded if more than 25% of participants were studied with a high risk of bias); inconsistency of results (downgraded if there was significant heterogeneity through inspection or if the I^2^ value was greater than 50%); indirection (a generalization of results; downgraded if more than 50% of participants were outside the target group); imprecision (downgraded if less than 400 participants were included in the comparison for continuous data) and other factors (for example, reporting bias or publication bias). If a study only evaluated the activity of a biomarker at a given time point, we considered the data inconsistent and imprecise (less than 400 participants for continuous outcomes and less than 300 participants for dichotomous outcomes), providing “low-certainty evidence,” which could be downgraded to “very low-certainty evidence” if we identified further limitations in relation to the risk of bias [25,34].

The overall certainty of evidence was defined as high certainty (further research is very unlikely to change our confidence in the estimate of effect), moderate certainty (further research is likely to have an important effect on our confidence in the estimate of effect and may change the estimate), low certainty (further research is very likely to have an important effect on our confidence in the estimate of effect and is likely to change the estimate), and very low certainty (very uncertain about the estimate) [34].

## 3. Results

### 3.1. Identification and Characteristics of the Included Studies

**Figure 1:**
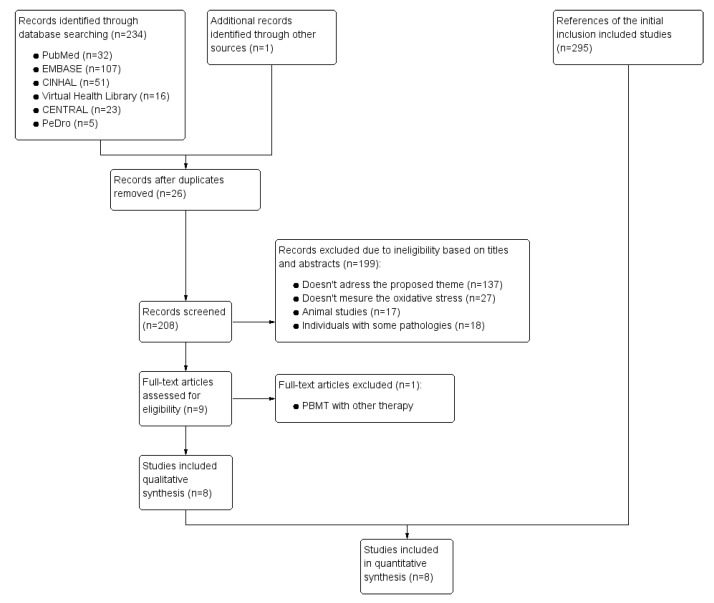
Flow of trials through the review.

The search strategy yielded 234 articles, among which nine studies were considered potentially relevant and were recovered for detailed analysis. We included eight randomized controlled trials (Figure 1), with a total number of 140 participants between 18 and 33 years of age. These studies were published between 2011 and 2022. Six trials included studies that performed crossover designs [35,36,37,38,39,40], and two were parallel trials [41,42]. The characteristics of the study can be found in Table 1.

### 3.2. Photobiomodulation Therapy (PBMT)

PBMT parameters and application of all studies are shown in Table 2. Only one article performed a protocol of exercises applying PBMT on the upper limbs [41]; the other authors performed a protocol on the lower limbs [35,36,37,38,39,40,42]. Seven articles [35,36,37,38,39,40,42] carried out the application of PBMT before exercising (3 min and 40 min); and two studies [40,41] were carried out from 2 min after exercise.

### 3.3. Characteristics of the Exercise Protocols

Authors proposed exercises involving a progressive running protocol on a motor-driven treadmill [35,38] and concentric/eccentric isokinetic contractions performed in the isokinetic dynamometer [41,42]. De Marchi et al. [36] performed video analysis to quantify the time and distance covered during a futsal match of 40 min. Jówko et al. [37] performed 45 isometric contractions associated with transcutaneous neuromuscular electrical stimulation (NMES) in the quadriceps muscle. Pinto et al. [40] submitted their participants to CrossFit^®^ training sessions, consisting of a sequence (21 repetitions, 15 repetitions and 9 repetitions) of three exercises (pedalling the Assault AirBike^®^, Hang Squat Clean and Box Jump Over). Meanwhile, Leal-Junior et al. [39] performed the Wingate test on a cycle ergometer at maximum velocity for 30 s, using a load equivalent to 7.5% of athlete body mass.

### 3.4. Oxidative Damage and Antioxidant Biomarkers

The most frequent oxidative stress biomarkers were carbonylated proteins (CP), damage to lipids [malondialdehyde (MDA) and thiobarbituric acid reactive substances (TBARS)], in 75% and in all studies, respectively. The catalase (CAT) was analyzed for four studies (50%) [38,40,41,42], and superoxide dismutase (SOD) for five trials [35,37,38,40,42]. Other biomarkers measured were glutathione peroxidase (GPx) and total antioxidant capacity (TAC) [37]. The time-points analyzed after exercise in the articles reporting trials were: 100% (*n* = 7) immediately after physical exercise (0–5 min) [35,36,37,38,39,40,41,42]; 38% (*n* = 3) 1 h after physical exercise [35,40,42]; 50% (*n* = 4) 24 h after [37,40,41,42]; 63% (*n* = 5) 48 h after [36,37,41,42]; 38% (*n* = 3) 72 h after [37,41,42]; and only 25% (*n* = 2) 96 h after exercise [37,42].

### 3.5. Risk of Bias

The studies evaluated the risk of bias through the PEDro scale. All included studies were considered excellent quality methodologically, with low risk of bias with scores varying from eight to ten points (mean was 9.13 points). The complete score of each of the studies is presented in Table 3. From the analysis of the PEDro scale, it was found that all articles met the criteria for sample selection, allocation, randomization, sample homogeneity, data analysis, and statistics (criteria 1 to 4, and 9 to 11). On the other hand, criteria 5 to 8 were not filled in in all the articles; some trials did not report or make it clear if the design was blinded with respect to the participants [37,41], sport therapists [37,39,41] or evaluators [35], and whether or not there was a sample loss [35].

### 3.6. Meta-Analysis

#### 3.6.1. Antioxidant Activity

##### Superoxide Dismutase (SOD)

**Figure 2:**
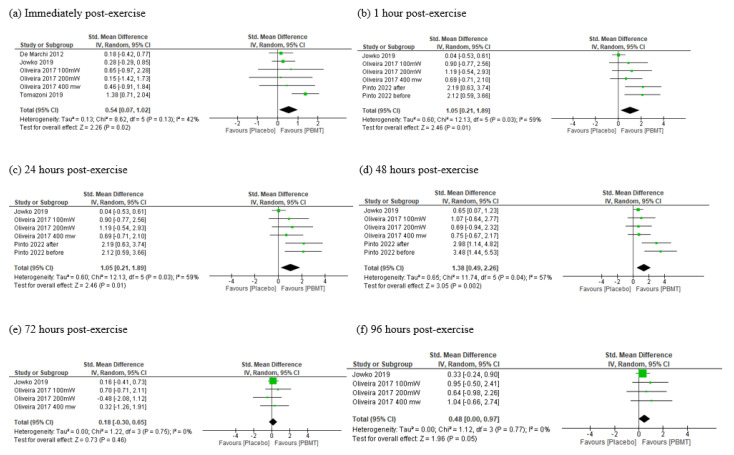
Meta-analysis of SOD activity immediately (**a**), 1 h (**b**), 24 h (**c**), 48 h (**d**), 72 h (**e**) and 96 h (**f**) post exercise. PBMT—photobiomodulation, IV—inverse variance, CI—confidence interval.

The antioxidant activity was measured through SOD in five trials [35,37,38,40,42] with different time-points (from immediately to 96 h post exercise). These pooled trials provided low to moderate certainty of evidence (downgraded due to inconsistency and/or imprecision) to suggest that PBMT can modulate SOD activity. We found significant effect in favor of PBMT immediately after exercise (SMD = 0.54, CI 95% 0.07 to 1.02, I^2^ = 42%, *p* = 0.02, see Figure 2a), 24 h after (SMD = 1.05, CI 95% 0.21 to 1.89, I^2^ = 59%, *p* = 0.01, see Figure 2c), 48 h after (SMD = 1.38, CI 95% 0.49 to 2.26, I^2^ = 57%, *p* = 0.002, see Figure 2d) and 96 h post exercise (SMD = 0.48, CI 95% 0.00 to 0.97, I^2^ = 0%, *p* = 0.05, see Figure 2f), but not at other time-points (1 and 72 h; see Figure 2b,e, respectively).

##### Catalase (CAT)

**Figure 3:**
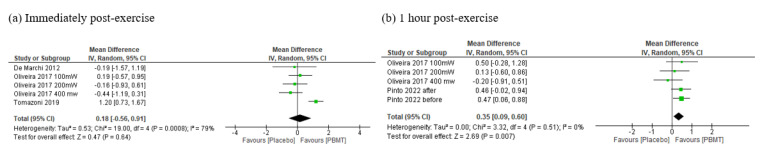
Meta-analysis of CAT activity immediately (**a**) and 1 h (**b**) post exercise. PBMT—photobiomodulation, IV—inverse variance, CI—confidence interval.

Only four trials [35,38,40,42] with low risk of bias compared the PBMT versus placebo and were meta-analyzed to estimate the effect on CAT activity post exercise. The meta-analysis provides low certainty of evidence (downgraded due to inconsistency and imprecision) that PBMT did not change the CAT activity (MD = 0.18 CI 95% −0.56 to 0.91, I^2^ = 79%, *p* = 0.64, see Figure 3a) immediately post exercise. There was moderate certainty of evidence (downgraded due to imprecision) indicating a significant effect in favor of PBMT in CAT activity one hour post exercise (MD = 0.35, CI 95% 0.09 to 0.50, I^2^ = 0%, *p* = 0.007, see Figure 3b). Although low certainty evidence (downgraded due to imprecision and inconsistency) showed that PBMT modulates CAT activity after exercise compared with placebo 24 and 48 h post exercise [40,42], we did not combine the results for the meta-analysis due to high heterogeneity (I^2^ > 90%), but we did report them descriptively (see Table 4). The analysis of remaining time points (72 and 96 h post exercise) was verified by one trial [42] with different PBMT power values. Only one group (100 mW) demonstrated an increased CAT activity (Table 4).

##### Glutathione Peroxidase (GPx) and Total Antioxidant Capacity (TAC)

One trial [37] with a low risk of bias provided a low certainty of evidence (downgraded due to inconsistency and imprecision) that PBMT has a similar effect to placebo therapy in TAC and GPx at all time-points (see Table 4).

#### 3.6.2. Oxidative Damage in Biomolecules

##### Oxidative Damage to Lipids

**Figure 4:**
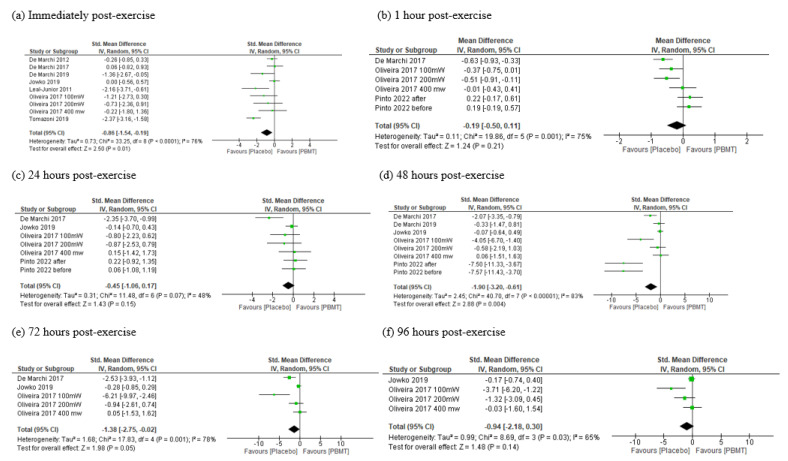
Meta-analysis damage to lipids immediately post exercise (**a**) and 1 h (**b**), 24 h (**c**), 48 h (**d**), 72 h (**e**) and 96 h (**f**) post exercise. PBMT—photobiomodulation, IV—inverse variance, CI—confidence interval.

Based on seven studies [35,36,37,38,39,41,42], there was low certainty of evidence (downgraded due to imprecision and inconsistency) that PBMT could reduce lipid damage immediately after exercise (SMD = −0.72, CI 95% −1.42 to −0.02, I^2^ = 77%, *p* = 0.04, see Figure 4a). We observed that a single PBMT session did not decrease lipid damage levels after one hour of exercise (MD = −0.19, 95% CI −0.50 to 0.12, I^2^ = 75%, *p* = 0.22, see Figure 4b), and 24 h (SMD = −0.42, 95% CI −1.04 to 0.19, I^2^ = 48%, *p*= 0.18, see Figure 4c). Based on low certainty of evidence (downgraded due to imprecision and inconsistency), the PBMT reduced the damage to lipids 48 h (SMD = −1.84, 95% CI −3.10 to −0.57, I^2^ = 82%, *p* = 0.004, see Figure 4d) and 72 h post exercise (SMD = −1.38, 95% CI −2.75 to −0.02, I^2^ = 78%, *p*= 0.05, see Figure 4e). However, from 96 h after exercise, PBMT showed no decrease in oxidative damage to lipids (SMD =−0.94, 95% CI −2.18 to 0.30, I^2^ = 65%, *p* = 0.14, see Figure 4f).

##### Oxidative Damage to Proteins

**Figure 5:**
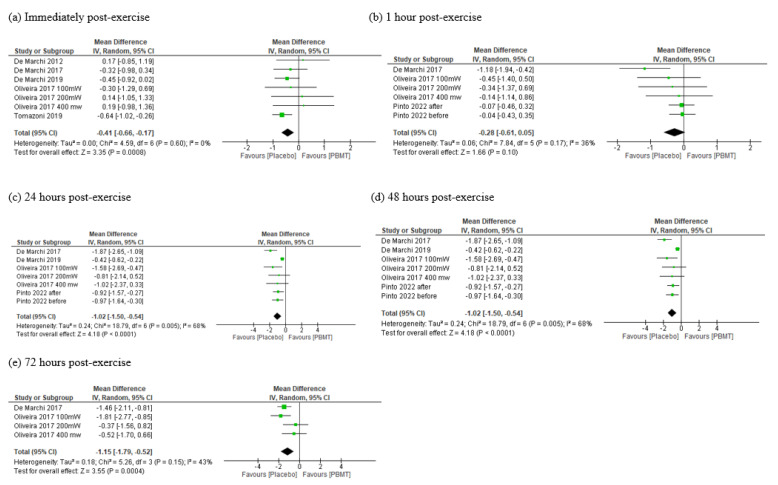
Meta-analysis of damage to proteins immediately post exercise (**a**) and 1 h (**b**), 24 h (**c**), 48 h (**d**) and 72 h (**e**) post exercise. PBMT—photobiomodulation, IV—inverse variance, CI—confidence interval.

Five studies [35,36,38,41,42] provided moderate certainty of evidence (downgraded due to imprecision) that PBMT reduced protein damage post exercise (MD = −0.41 CI 95% −0.65 to −0.16, I^2^ = 0%, *p* = 0.001, see Figure 5a). However, from one hour post exercise, we did not find any difference between PBMT and placebo (MD = −0.28 CI 95% −0.61 to 0.05, I^2^ = 36%, *p* = 0.10, see Figure 5b). At the other meta-analyzed time points, PBMT was shown to be able to reduce protein damage 24 h (MD = −0.97, CI 95% −1.30 to −0.64, I^2^ = 0%, *p*< 0.00001, see Figure 5c), 48 h (MD = −1.01, CI 95% −1.48 to −0.53, I^2^ = 67%, *p*< 0.0001, see Figure 5d) and 72 h (MD = −1.15, CI 95% −1.79 to −0.52, I^2^ = 43%, *p* = 0.0004, see Figure 5e) post exercise. Only one trial [39] verified the PC concentration at 96 h post exercise with positive effects in favor of PBMT, and we describe these findings in Table 4.

### 3.7. Certainty of Evidence

The overall evidence for each time-point in each comparison can be seen in Table S2 (see Supplementary Materials). The certainty of evidence for antioxidant and oxidative stress outcomes was subdivided according to plasma markers of redox balance (i.e., SOD, CAT, TBARS, etc.). In general, there is evidence of low certainty (downgraded due to inconsistency and imprecision) to moderate certainty (downgraded due to imprecision) of evidence that PBMT can reduce oxidative damage and modulate the antioxidant activity.

## 4. Discussion

In this review, clinical trials that assessed the effects of PBMT on exercise-induced oxidative stress in healthy individuals were summarized, comparing its results with placebo/sham therapy. Our findings indicated that PBMT was able to minimize the damage to lipids and proteins induced by exercise. However, the effect of PBMT on lipid damage seems to be related to the exercise protocol (anaerobic or aerobic) to which the individual is submitted and the magnitude of the damage caused. Moreover, PBMT increased the enzymatic antioxidant activity in SOD and CAT, but not for GPx. This effect size seems to be increased in subjects exposed to high intensity training more than in healthy individuals (physically active or untrained men). Furthermore, the analysis of one of the trials demonstrated that PBMT was not able to change TAC levels in the short-term.

We observed that the vast majority of included studies (86%) used the application of PBMT before exercising. Other systematic reviews [23,24] presented similar data, where in 92% and 67% of the included clinical trials, the application was before exercising. Regardless of whether the application is carried out before [35,36,37,38,39,40,42] or after [40,41] the exercise, our analysis showed that PBMT was able to minimize oxidative damage. Although PBMT irradiation is applied at different times, it seems that the mechanisms of action by which the effects of light modulate redox metabolism are not distinct [43]. Recently in vitro studies demonstrated that the application of PBMT promoted a protective effect in muscle cells exposed to myotoxic agents [44]. Based on this evidence, it is possible that PBMT applied before exercise provides a cellular protective effect, contributing to the antioxidant system to combat and control the production of ROS induced by intense exercise, reducing damage to muscle tissue. Meanwhile, the application of PBMT post exercise was able to increase the mitochondrial membrane potential towards normal levels [45] and lower the production of ROS or act as ROS sweeper [46]. Our findings provided moderate to low certainty of evidence (downgraded due to imprecision and/or inconsistency) that PBMT has a potential antioxidant effect, which could be a key mechanism of action that contributes to the ergogenic effects of PBMT. It may partially explain the increase in performance, longer time to exhaustion, greater number of repetitions, and maintenance of muscle strength post exercise promoted by PBMT described in the literature [24,25].

Currently, it is known that the production of ROS, as superoxide radical (O_2_^•^−) and hydrogen peroxide (H_2_O_2_), occurs predominantly by the contraction of skeletal muscles performed during physical exercise [40,41]. Andrade et al. [47] demonstrated that more prolonged exposure to H_2_O_2_ results in strength decline, and that this occurs due to the cytotoxicity of H_2_O_2_ which is capable of forming the hydroxyl radical (·OH). The hydroxyl radical is highly reactive, has a strong oxidizing potential and is capable of damaging the muscle cell structure, resulting in lipid peroxidation and protein carbonylation [48]. However, we observed in this review that PBMT decreased the levels of oxidative damage markers in healthy subjects post exercise when compared to the placebo condition, thus indicating that PBMT can modulate ROS production. Accordingly, studies conducted in vitro [49,50] identified that PBMT modulated ROS generation, activating redox-sensitive transcription factors such as NF-kB, thus improving cellular antioxidant capacity.

The first line of defense to oxidative damage is represented by the action of endogenous antioxidants (SOD, CAT and GPx), whose main role is to maintain redox homeostasis. Based on pooled studies with a low risk of bias [35,37,38,40,42], our results showed that PBMT improved or maintained SOD activity post exercise, this enzyme being responsible for the dismutation of O_2_•−, thus generating H_2_O_2_. In turn, the H_2_O_2_ generated is dismutated by the action of CAT or GPX. The activity of these enzymes varies in relation to the concentration of H_2_O_2_, with CAT being more effective at high concentrations of H_2_O_2_, and GPx at low concentrations of H_2_O_2_ [5]. We observed that in the exercise protocol that involved both aerobic and anaerobic effort, the application of PBMT increased CAT activity [42]; while in the anaerobic effort protocol alone, PBMT increased neither CAT nor GPX activity [37,42]. Therefore, we suggest that the effects of PBMT on enzymatic antioxidant activity (i.e., SOD, CAT and GPx) may be related to the magnitude of alteration in redox homeostasis caused by exercise. A recent review [51] supports this hypothesis, as it showed that PBMT promotes better results when exercise causes greater muscle damage in the exercised muscle.

Based on our results and current literature [25,26,52], PBMT provides cytoprotective effects on skeletal muscle. This occurs through improvement in mitochondrial function by the interaction of light with cytochrome oxidase, regulation of the potential of the mitochondrial membrane, and modulation of the density and viscosity of the nanoscopic layer of interfacial water [53,54,55,56,57,58], all processes stimulate and enhance cell metabolism. Some authors also report that PBMT can provide higher levels of cellular respiration and ATP synthesis due to the modulation of aerobic metabolism enzymes, such as complexes I, II, III, and IV of the mitochondrial respiratory chain [19,20,59,60,61,62]. Although in our meta-analysis, we have not seen a significant increase (*p* > 0.05) in the activities of antioxidant enzymes at certain time-points (e.g., in SOD activity 72 h post exercise), most laboratory studies of PBMT which examined various types of animal models, diseases, or injuries, as well as exercises, observed the modulation of PBMT on antioxidant enzymes [19,20,43,57,58,59]. However, the sense of modulation (increase or decrease in activity or enzyme concentration) seems to be related to several factors, such as types of protocols (injuries, diseases, exercises), parameters and/or application of PBMT, generation of reactive species, inflammatory processes, types of fibers, exercise intensity, and muscle damage, among others. Corroborant with our findings in this review, other authors suggest that PBMT can act as a modulator of antioxidant enzymes and also as scavengers of radicals, depending on the metabolic condition that is presented [60].

PBMT has a dose-response effect, so it is important to identify what doses are used for the proposed objective [61]. In general, the studies included used the total energy dose for small muscles (biceps brachii and triceps sural) from 41.7 J to 100 J. For large muscles (quadriceps femoris and hamstrings), the dose varied from 120 to 450 J. The recently published guidelines with clinical and scientific recommendations for the use of PBMT for exercise performance enhancement and post-exercise recovery recommend a dose range from 20 J to 60 J for small muscle groups, and from 120 J to 300 J for large muscle groups [63]. It was observed that the majority of the studies included [35,36,37,39,40,41,42] are within the recommendations. Only the clinical trial conducted by Tomazoni et al. [38] did not follow the recommendations described by Leal-Junior et al. [63], with an energy dose of 100 J to a small muscle group and 450 J to a large muscle group. However, the parameters used by the authors [38] demonstrated positive effects in modulating oxidative stress, very likely due to the previous optimization of dose [63] and power [42] using this very same device. On the other hand, Jowko et al. [37], using the parameters recommended for PBMT [63], had no positive effects on the modulation of the redox state. Thus, its results were different from the other studies included [35,38,40]. Compared to baseline conditions, the oxidative damage generated in the placebo group was significant greater (*p* < 0.05), but between interventions (PBMT versus placebo) there was no statistically significant difference for lipid damage at any time-point [37]. Therefore, due to the wide variability of features from the different devices commercially available, it is stressed that the optimization of every device/technology is paramount to achieving positive effects, despite the discrepancy in the current recommended dose ranges/energy for both large and small muscle groups [24,63].

It is known that the level of oxidative damage can be influenced by the type, time, duration of exercise, age, level of physical activity, nutritional status, and antioxidant capacity of each individual [6,13,14,15]. The participants in our analysis consisted of healthy male athletes, physically active participants and non-active individuals. Recent evidence indicates that training status can influence oxidative damage responses and adaptations induced by exercise [13,62]. This adaptive effect on exercise-induced redox metabolism (increased antioxidant enzymatic defenses) can generate an increase in resistance to oxidative stress, that is, lower damage to biomolecules [5]. This may explain the heterogeneity found in some time-points, represented by inconsistency (I^2^ > 50%). Among the studies included in this review, studies carried out with athletes showed a greater effect size [38,40,42] and longer duration of antioxidant activity (up to 96 h after exercise) [42] promoted by PBMT. Furthermore, this suggests that PBMT can enhance the responses of the antioxidant defense system in a single session of therapy, but whether these acute effects provided by PBMT are potentiated with a long-term protocol (>4 weeks) on redox metabolism is still an obscure topic in the literature. On the other hand, current studies demonstrate that PBMT can be an important tool in preventing muscle fatigue, improving performance, and also decreasing atrophy [63,64]. In a recently published study by our research group, we observed clinical improvement and decreased diaphragmatic muscle atrophy in ICU intubated patients when they received a daily application of PBMT [16]. Therefore, studies that investigate these effects in fatigue-related pathologies, such as chronic fatigue syndrome and LONG-COVID-19, and others, are warranted.

To the authors’ knowledge, this is the first systematic review with meta-analysis to investigate the effects of PBMT on exercise-induced oxidative stress in humans. Its strengths are related to a comprehensive search in several databases. The search, without language and year restriction, included an evaluation of methodological quality by the PEDro Scale, the use of GRADE system to assess the certainty of evidence and a prospectively registered protocol. Performing a statistical analysis should also be considered as a strong point since currently, the relationship between oxidative stress and PBMT is a field that few researchers have investigated.

However, this review is not free of limitations. We observed inconsistency or imprecision in some comparisons due to the low number of trials and small sample size (>10 volunteers per group) in some studies (despite reporting the calculation of the sample size). Second of all, the various healthy populations have been mixed, and future studies should be conducted to investigate whether there is a difference in the effect of PBMT on redox metabolism between specific populations (e.g., trained versus untrained and/or athletes versus non-athletes). In addition, our results have important implications for the practitioners of sports and musculoskeletal rehabilitation, since PBMT can modulate oxidative stress and maintain redox homeostasis which can contribute to shorter muscle recovery time, minimize the deficits in the ability to produce strength after exercise and reduce induced muscle damage by exercise.

## 5. Conclusions

In conclusion, our results are based on the low and moderate certainty of evidence with studies of good to excellent methodological quality according to the PEDro scale, suggesting that PBMT is an effective approach to enhance antioxidant activity and minimize exercise-induced oxidative damage. Therefore, we encourage future studies to be carried out with a high methodological quality to identify the effect of PBMT in modulation of oxidative stress in the long-term in aerobic and anaerobic training programs.

## Figures and Tables

**Table 1:** Characteristics of included trials.

	De Marchi et al. [35]	De Marchi et al. [36]	Jówko et al. [37]	Tomazoni et al. [38]	Leal-Junior et al. [39]	Pinto et al. [40]	De Marchi et al. [41]	Oliveira et al. [42]
Design	CRDB	CRTB	CRDB	CRTB	CRDB	CRTB	RDB	RDB
Participants (N sample, age, sex)	Healthy untrained volunteers (*n* = 22, 22.02 ± 3.02 years, male)	Futsal athletes (*n* = 6, 26.16 ± 6.91 years, male)	Moderately active healthy (*n* = 24, 21.9 ± 0.3 years, male)	Soccer players (*n* = 22, 18.85 ± 0.61 years, male)	Volleyball athletes (*n* = 6, 18.57 ± 0.98 years, male)	CrossFit athletes (*n* = 12, 27 ± 5.54 years, male)	Healthy physically active (*n* = 20, 25.30 ± 3.32 years, male)	Soccer players (*n* = 28, 18.62 ± 0.73 years, male)
Exercise Protocol	Progressive running protocol on a motor-driven treadmill	Matches of 40 min	45 electrically evoked tetanic, isometric contractions of the quadriceps femoris	Progressive running protocol on a motor-driven treadmill	Wingate test performed on cycle ergometer with maximum velocity during 30 s using load of 7.5% of body mass	WOD was composed of three series of Assault AirBike^®^, Hang Squat Clean, and Box Jump Over exercises with 21, 15 and 9 repetitions	Fatigue-inducing protocol on the isokinetic dynamometer	75 eccentrics isokinetic contractions of the knee extensors of the nondominant leg
Oxidative damage markers	TBARS, CP	TBARS, CP	MDA *	TBARS, CP	TBARS	TBARS, CP	TBARS, CP	TBARS, CP
Antioxidant capacity markers	SOD, CAT	-	GPx, SOD, TAC	SOD, CAT	-	SOD, CAT	-	SOD, CAT
Time points evaluated	Immediately post exercise	Immediately and 48 h post exercise	Immediately, 24, 48, 72 and 96 h post exercise	Immediately post exercise	Immediately post exercise	1, 24 and 48 h post exercise	Immediately, 1, 24, 48 and 72 h post exercise	Immediately, 1, 24, 48, 72 and 96 h post exercise

CRDB—cross-over randomized double-blind, RDB—randomized double-blind, CRTB—cross-over triple blind, TBARS—Thiobarbituric acid reactive substances, CP—oxidative damage to protein, SOD—superoxide dismutase, CAT—catalase, MDA—malondialdehyde, GPx—Glutathione peroxidase, TAC—Total Antioxidant Capacity. * The TBARS test measures the MDA present in the sample.

**Table 2:** Photobiomodulation therapy parameters.

	De Marchi et al. [35]	De Marchi et al. [36]	Jówko et al. [37]	Tomazoni et al. [38]	Leal-Junio et al. [39]	Pinto et al. [40]	De Marchi et al. [41]	Oliveira et al. [42] *
Number of diodes	5	12 (4 super-pulsed infrared, 4 red LEDs, 4 Infrared LEDs)	Cluster of 4 semiconducting lasers	5	69 (34 red diodes and 35 infrared diodes)	20 ( 4 lasers, 8 red LED’s, 8 infrared LED’S)	69 (34 red LEDs and 35 infrared LEDs)	5
Wavelength (nm)	810 (infrared)	905 (± 1) super-pulsed infrared, 640 (±10) red, 875 (±10) infrared	830 nm	810 nm (infrared)	660 nm (red) and 850 nm (infrared)	905 nm (laser), 633 nm (red LED’s), 850 nm (infrared LED’s)	660 (red) and 850 (infrared)	810 nm (infrared)
Frequency (Hz)	Continuous output	250 super-pulsed infrared, 2 red, 16 infrared	Continuous output	Continuous output	Continuous output	250 laser, 2 red LED’s, 250 infrared LED’s	Continuous output	Continuous output
Optical output (mW)	200 each diode	1.25 super-pulsed infrared, 60 red, 70 infrared	200	100 each diode	10 (red) and 30 (infrared)	1.25 laser, 25 red LED’s, 40 infrared LED’s	10 (red) and 30 (infrared)	100 (a), 200 (b), 400 (c)
LED spot size (cm^2^)	0.0364 each spot, total 0.182	0.44 each super-pulsed infrared, 0.9 each red, 0.9 each infrared	Not reported	0.0364 each spot, total 0.182	0.2 (for both red and infrared)	0.32 each laser, 0.85 each red LED’s, 0.56 each infrared LED	0.2 (red and infrared), total spot sizes 13.8	0.0364 each spot, total 0.182
Power density (W/cm^2^)	5.495 each spot	0.071 super-pulsed infrared, 1.66 red, 1.94 infrared (each)	Not reported	2.75 each spot	0.05 (for red) and 0.15 (for infrared)	3.91 each laser, 29.41 each red LED’s, 71.23 each infrared LED	0.05 (for red) and 0.15 (for infrared)	2.75 (a), 5.50 (b), 11.00 (c)
Energy (J)	30 on each point (6 for each spot)	30 (0.285 super-pulsed infrared, 13.68 red, 15.96 infrared).	30	50 (10 each diode)	41.7 on each point (0.3 for each red and 0.9 for each infrared)	0.16 each laser, 3.22 each red LED’s, 5.16 each infrared LED	41.7 (0.3 from each red LED, 0.9 from each infrared LED)	50 (10 each diode)
Energy density (J/cm^2^)	164.85 (for each laser spot)	0.162 super-pulsed infrared, 3.8 red, 4.43 infrared (each)	Not reported	275 (for each laser spot)	1.5 on each point (for red) and 4.5 (for infrared)	0.50 each laser, 3.79 each red LED’s, 9.21 each infrared LED	1.5 (for red) and 4.5 (for infrared)	275 (for each laser spot)
Treatment time (sec)	30 on each point	228 on each point	Not reported	100 on each point	30	129 s knee extensors and 115 s knee flexors and plantar flexors	30	100 (a), 50 (b), 25 (c)
Number of irradiation points per muscle	6 sites in quadriceps, 4 points in hamstrings and 2 points in gastrocnemius	6 points in quadriceps, 6 points in hamstrings and 2 points in gastrocnemius	6 sites in quadriceps (2 medial, 2 lateral and 2 central)	9 points in quadriceps, 6 points in hamstrings and 2 points in gastrocnemius	2 points in quadriceps bilaterally	4 sites on knee extensor, 3 sites on knee flexor and 1 site on plantar flexor	1 (biceps brachii)	6 sites in quadriceps (2 medial, 2 lateral and 2 central)
Total energy delivered per muscle (J)	120 in hamstrings and 60 in others muscles	180 in hamstrings and 90 in others muscles	180 in knee extensor	450 knee extensor, 300 knee flexor, and 100 plantar flexor	83.4	270 on knee extensor, 180 on knee flexor and 60 on plantar flexor	41.7	300 in knee extensor
Cluster area (cm^2^)	9.6	20	25	9.6	13.8	33	13.8	9.6
Magnetic field (mT)	-	35	-	-	-	110	-	-
Application mode	Cluster held stationary in skin contact with a 90° angle and slight pressure	Cluster held stationary in skin contact with a 90° angle and slight pressure	Cluster held stationary in skin contact with a 90° angle and slight pressure (0° angle of incidence)	Cluster in stationary position with slight pressure and direct contact with skin	Cluster held stationary in skin contact with a 90° angle and slight pressure	Direct skin contact and slight pressure	Cluster held stationary in skin contact with a 90° angle and slight pressure	Cluster in stationary position with slight pressure in direct contact with skin over quadriceps with volunteer seated, knee at rest with 90° of flexion

* Different letters in the same study mean groups with different PBMT parameters.

**Table 3:** Risk of Bias (PEDro scale).

Scores	De Marchi et al. [35]	De Marchi et al. [36]	Jówko et al. [37]	Tomazoni et al. [38]	Leal-Junior et al. [39]	Pinto et al. [40]	De Marchi et al. [41]	Oliveira et al. [42]
1	Y	Y	Y	Y	Y	Y	Y	Y
2	Y	Y	Y	Y	Y	Y	Y	Y
3	Y	Y	Y	Y	Y	Y	Y	Y
4	Y	Y	Y	Y	Y	Y	Y	Y
5	Y	Y	N	Y	Y	Y	N	Y
6	Y	Y	N	Y	N	Y	N	Y
7	N	Y	Y	Y	Y	Y	Y	Y
8	N	Y	Y	Y	Y	Y	Y	Y
9	Y	Y	Y	Y	Y	Y	Y	Y
10	Y	Y	Y	Y	Y	Y	Y	Y
11	Y	Y	Y	Y	Y	Y	Y	Y
**TOTAL**	**8**	**10**	**8**	**10**	**9**	**10**	**8**	**10**

N—no, Y—yes.

**Table 4:** Mean difference and 95% confidence interval between changes of PBMT versus placebo at time-points that were not meta-analyzed.

Biomarker	Study	PBMT Groups	Immediately Post	1 h	24 h	48 h	72 h	96 h
CAT	Oliveira et al. [42]	PBMT 100 mW	-	-	0.34 [−0.12, 0.80]	0.58 [0.16, 1.00] *	0.49 [0.03, 0.95] *	0.62 [0.10, 1.14] *
		PBMT 200 mW	-	-	−0.14 [−0.56, 0.28]	0.20 [−0.16, 0.56]	0.09 [−0.29, 0.47]	0.03 [−0.38, 0.44]
		PBMT 400 mW	-	-	0.26 [−0.14, 0.66]	0.68 [0.29, 1.07] *	0.26 [−0.18, 0.70]	0.44 [−0.00, 0.88]
	Pinto et al. [40]	PBMT after	-	-	1.69 [1.34, 2.04] *	1.92 [1.65, 2.19] *	-	-
		PBMT before	-	-	1.61 [1.27, 1.95] *	2.00 [1.74, 2.26] *	-	-
GPx	Jówko et al. [37]	-	−1.8 [−12.58, 8.98]	-	−7.6 [−19.41, 4.21]	4.8 [−8.51, 18.11]	−4.8 [−16.29, 6.69]	−8.6 [−19.86, 2.76]
TAC	Jówko et al. [37]	-	−0.15 [−0.31, 0.02]	-	−0.12 [−0.27, 0.03]	−0.1 [0.26, 0.06]	−0.06 [−0.22, 0.10]	0 [−0.14, 0.14]
Damage to protein	Oliveira et al. [42]	PBMT 100 mW	-	-	-	-	-	−1.48 [−2.20, −0.76] *
		PBMT 200 mW	-	-	-	-	-	−0.42 [−1.15, 0.31]
		PBMT 400 mW	-	-	-	-	-	−0.93 [−1.73, −0.13] *

GPx—glutathione peroxidase, TAC—Total Antioxidant Capacity, CAT—catalase, * values are mean and 95% Confidence Interval, *—significant effect in PBMT group. Different letters (a, b and c) in the same study indicate the PBMT groups with no similar parameters.

## Supplementary Materials

The following supporting information can be downloaded at: www.mdpi.com/article/10.3390/antiox11091671/s1, Search Strategy S1, Table GRADE S2.
