# Supplementary material for: Can Photobiomodulation Therapy (PBMT) Minimize Exercise-Induced Oxidative Stress? A Systematic Review and Meta-Analysis
Source: Antioxidants (Basel). 2022 Aug 27;11(9):1671. doi: 10.3390/antiox11091671 (PMC9495825; doi:10.3390/antiox11091671)
Supplement: Supplementary file 1 [file antioxidants-11-01671-s001.zip › Supplementary 1 PBMT in oxidative stress - Antioxidants.pdf]

Supplementary 1. Search Strategy

PUBMED (Advanced Search)

#1 Photobiomodulation
#2 Phototherapy
#3 Low-level light therapy
#4 Low-level laser therapy
#5 Low intensity power therapy
#6 Light-emitting diode
#7 Laser Phototherapy
#8 Low-Power Laser
#9 #1 OR #2 OR #3 OR #4 OR #5 OR #6 OR #7 OR #8
#10 Oxidative stress
#11 Oxidative Stresses
#12 Anti-oxidative Stress
#13 Oxidative Damage
#14 Oxidative Stress Injury
#15 #10 OR #11 OR #12 OR #13 OR #14
#16 Randomized controlled trial
#17 Controlled clinical trial
#18 Comparative study
#19 random.
#20 placebo
#21 trial.
#22 groups
#23 #16 OR #17 OR #18 OR #19 OR 20 OR #21 OR #22
#24 Exercise
#25 Physical exercise
#26 Acute Exercise
#27 Isometric exercise
#28 Aerobic exercise
#29 Exercise Training
#30 #24 OR #25 OR #26 OR #27 OR #28 OR #29
#31 #9 AND #15 AND #23 AND #30

EMBASE (QUICK; All Fields)

#1 photobiomodulation OR phototherapy OR (low-level AND light AND therapy) OR (low-level AND laser AND therapy) OR (laser AND phototherapy) OR (low AND intensity AND power AND therapy)

#2 oxidative AND stress OR (oxidative AND stresses) OR 'anti-oxidative stress' OR ('anti oxidative' AND ('stress'/exp OR stress)) OR 'oxidative damage'/exp OR 'oxidative damage' OR (oxidative AND damage) OR 'antioxidant'/exp OR antioxidant

#3 exercise OR (physical AND exercise) OR (acute AND exercise) OR (isometric AND exercise) OR 'resistance training' OR (aerobic AND exercise)

#4 #1 AND #2 AND #3

CINAHL (Ebsco)

Advanced Search

S1[All text] Photobiomodulation therapy OR Low level laser therapy OR Phototherapy OR Low intensity power therapy

S2 [All text] Oxidative stress OR Oxidative Stresses OR Anti-oxidative Stress OR Oxidative Damage OR Oxidative Stress Injury

S3 [All text] Exercise OR Physical exercise OR Acute Exercise OR Isometric exercise OR Aerobic exercise OR Exercise Training

S4 S1 AND S2 AND S3

Virtual Health Library (VHL)

Advanced Search "Title, Abstract, subject"

Photobiomodulation OR phototherapy OR (low-level AND light AND therapy) OR (low-level AND laser AND therapy) OR (laser AND phototherapy) OR (low AND intensity AND power AND therapy)) AND (oxidative AND stress OR (oxidative AND stresses) OR 'anti-oxidative stress' OR ('anti oxidative' AND ('stress'/exp OR stress)) OR 'oxidative damage'/exp OR 'oxidative damage' OR (oxidative AND damage) OR 'antioxidant'/exp OR antioxidant) AND (exercise OR (physical AND exercise) OR (acute AND exercise) OR (isometric AND exercise) OR 'resistance training' OR (aerobic AND exercise)

Filter> Type of Study "Controlled clinical trial"

Cochrane Central Register of Controlled Trials (CENTRAL)

Advanced Search; Search Manager

#1 Photobiomodulation therapy OR Low level laser therapy OR Phototherapy OR Low intensity power therapy

#2 Oxidative stress OR Oxidative Stresses OR Anti-oxidative Stress OR Oxidative Damage OR Oxidative Stress Injury

#3 Exercise OR Physical exercise OR Acute Exercise OR Isometric exercise OR Aerobic exercise OR Exercise Training

#4 #1 AND #2 AND #3

"Box Trials"

PEDro

“Advanced Search”

Abstract & Title: Oxidative stress

Method: Clinical Trial

Title Only: Low level laser therapy OR Photobiomodulation
