# Supplementary material for: Can Photobiomodulation Therapy (PBMT) Minimize Exercise-Induced Oxidative Stress? A Systematic Review and Meta-Analysis
Source: Antioxidants (Basel). 2022 Aug 27;11(9):1671. doi: 10.3390/antiox11091671 (PMC9495825; doi:10.3390/antiox11091671)
Supplement: Supplementary file 1 [file antioxidants-11-01671-s001.zip › Supplementary 2 - GRADE-S2.pdf]

GRADE

1. PBMT compared with placebo PBMT in SOD activity (Immediately, 1, 24, 48, 72 and 96 hours after exercise)

Certainty assessment							№ of patients		Effect		Certainty
№ of studies	Study design	Risk of bias	Inconsistency	Indirectness	Imprecision	Other considerations	PBMT	Placebo	Relative (95% CI)	Absolute (95% CI)	
4	randomised trials	not serious	not serious	not serious	serious [b]	none	89	75	-	SMD **0.55 SD higher** (0.08 to 1.02 higher)	⊕⊕⊕◯ MODERATE
2	randomised trials	not serious	not serious	not serious	serious [b]	none	33	19	-	MD **0.13 nmol/ml higher** (0.14 lower to 0.40 higher)	⊕⊕◯◯ LOW
3	randomised trials	not serious	serious [a]	not serious	serious [b]	none	57	43	-	SMD 1.05 **SD higher** (0.21 lower to 1.89 higher)	⊕⊕◯◯ LOW
3	randomised trials	not serious	serious [a]	not serious	serious [b]	none	57	43	-	SMD 1.39 **SD higher** (0.49 to 2.26 higher)	⊕⊕◯◯ LOW
2	randomised trials	not serious	not serious	not serious	serious [b]	none	45	31	-	SMD **0.18 SD higher** (0.30 lower to 0.65 higher)	⊕⊕⊕◯ MODERATE
2	randomised trials	not serious	not serious	not serious	serious [b]	none	45	31	-	SMD **0.48 SD higher** (0.00 to 0.97 higher)	⊕⊕⊕◯ MODERATE

SOD: Superoxide Dismutase; **CI:** Confidence interval; **SMD:** Standardized mean difference; **SD:** Standard deviation.

Explanations

a. Forest plots shows severe inconsistency ($I^2 > 50\%$)

b. The total number of participants in this comparison is lower than optimal information size (n < 400)

2. PBMT compared with placebo PBMT in CAT activity (Immediately, 1, 24, 48, 72 and 96 hours after exercise)

Certainty assessment							№ of patients		Effect		Certainty
№ of studies	Study design	Risk of bias	Inconsistency	Indirectness	Imprecision	Other considerations	PBMT	Placebo	Relative (95% CI)	Absolute (95% CI)	
3	randomised trials	not serious	serious [a]	not serious	serious [b]	none	65	51	-	MD **0.18 U CAT/mg of protein higher** (0.56 lower to 0.91 higher)	⊕⊕◯◯ LOW
2	randomised trials	not serious	not serious	not serious	serious [b]	none	21	7	-	MD **0.37 U CAT/mg of protein higher** (0.11 to 0.62 higher)	⊕⊕⊕◯ MODARATE
2	randomised trials	not serious	serious [a]	not serious	serious [b]	none	45	45	-	Not pooled	⊕⊕◯◯ LOW
2	randomised trials	not serious	serious [a]	not serious	serious [b]	none	45	45	-	Not pooled	⊕⊕◯◯ LOW
1	randomised trials	not serious	serious [c]	not serious	serious [c]	none	21	7	-	Not pooled	⊕⊕◯◯ LOW
1	randomised trials	not serious	serious [c]	not serious	serious [c]	none	21	7	-	Not pooled	⊕⊕◯◯ LOW

CAT: Catalase; **CI:** Confidence interval; **MD:** Mean difference

Explanations

a. Forest plots shows severe inconsistency ($I^2 > 50\%$)

b. The total number of participants in this comparison is lower than optimal information size (n < 400)

c. Single study in this comparison

3. PBMT compared with placebo PBMT in TAC activity (Immediately, 1, 24, 48, 72 and 96 hours after exercise)

Certainty assessment							№ of patients		Effect		Certainty
№ of studies	Study design	Risk of bias	Inconsistency	Indirectness	Imprecision	Other considerations	PBMT	Placebo	Relative (95% CI)	Absolute (95% CI)	
1	randomised trials	not serious	serious [a]	not serious	serious [a,b]	none	24	24	-	Not pooled	⨁⨁◯◯ LOW
1	randomised trials	not serious	serious [a]	not serious	serious [a, b]	none	24	24	-	Not pooled	⨁⨁◯◯ LOW
1	randomised trials	not serious	serious [a]	not serious	serious [a,b]	none	24	24	-	Not pooled	⨁⨁◯◯ LOW
1	randomised trials	not serious	serious [a]	not serious	serious [a,b]	none	24	24	-	Not pooled	⨁⨁◯◯ LOW
1	randomised trials	not serious	serious [a]	not serious	serious [a,b]	none	24	24	-	Not pooled	⨁⨁◯◯ LOW

TAC: Total Antioxidant Capacity; **CI:** Confidence interval;

Explanations

a. Single study in this comparison

b. The total number of participants in this comparison is lower than optimal information size (n <400)

4. PBMT compared with placebo PBMT in GPx activity (Immediately, 24, 48, 72 and 96 hours after exercise)

Certainty assessment							№ of patients		Effect		Certainty
№ of studies	Study design	Risk of bias	Inconsistency	Indirectness	Imprecision	Other considerations	PBMT	Placebo	Relative (95% CI)	Absolute (95% CI)	
1	randomised trials	not serious	serious [a]	not serious	serious [a,b]	none	24	24	-	Not pooled	⨁⨁◯◯ LOW
1	randomised trials	not serious	serious [a]	not serious	serious [a,b]	none	24	24	-	Not pooled	⨁⨁◯◯ LOW
1	randomised trials	not serious	serious [a]	not serious	serious [a, b]	none	24	24	-	Not pooled	⨁⨁◯◯ LOW
1	randomised trials	not serious	serious [a]	not serious	serious [a, b]	none	24	24	-	Not pooled	⨁⨁◯◯ LOW
1	randomised trials	not serious	serious [a]	not serious	serious [a, b]	none	24	24	-	Not pooled	⨁⨁◯◯ LOW

GPx: Glutathione peroxidase; **CI:** Confidence interval;

Explanations

a. Single study in this comparison

b. The total number of participants in this comparison is lower than optimal information size (n <400)

5. PBMT compared with placebo PBMT in damage to lipids (Immediately, 1, 24, 48, 72 and 96 hours after exercise)

Certainty assessment							№ of patients		Effect		Certainty
№ of studies	Study design	Risk of bias	Inconsistency	Indirectness	Imprecision	Other considerations	PBMT	Placebo	Relative (95% CI)	Absolute (95% CI)	
7	randomised trials	not serious	serious [a]	not serious	serious [b]	none	111	96	-	SMD **0.72 SD lower** (1.41 to 0.03 lower)	⨁⨁◯◯ LOW
3	randomised trials	not serious	serious [a]	not serious	serious [b]	none	41	27	-	MD **0.19 nmol/ml lower** (0.50 lower to 0.11 higher)	⨁⨁◯◯ LOW
4	randomised trials	not serious	not serious	not serious	serious [b]	none	65	51	-	SMD **0.45 SD lower** (1.06 lower to 0.17 higher)	⨁⨁⨁◯ MODERATE
5	randomised trials	not serious	serious [a]	not serious	serious [b]	none	71	57	-	SMD **1.90 SD lower** (3.20 to 0.61 lower)	⨁⨁◯◯ LOW
3	randomised trials	not serious	serious [a]	not serious	serious [b]	none	53	39	-	SMD **1.38 SD lower** (2.75 to 0.02 lower)	⨁⨁◯◯ LOW
2	randomised trials	not serious	serious [a]	not serious	serious [b]	none	45	31	-	SMD **0.94 SD lower** (2.18 lower to 0.30 higher)	⨁⨁◯◯ LOW

CI: Confidence interval; **SMD:** Standardized mean difference; **SD:** Standard deviation; **MD:** Mean difference

Explanations

a. Forest plots shows severe inconsistency ($I^2 > 50\%$)

b. The total number of participants in this comparison is lower than optimal information size (n < 400)

6. PBMT compared with placebo PBMT in damage to proteins (Immediately, 1, 24, 48, 72 and 96 hours after exercise)

Certainty assessment							№ of patients		Effect		Certainty
№ of studies	Study design	Risk of bias	Inconsistency	Indirectness	Imprecision	Other considerations	PBMT	Placebo	Relative (95% CI)	Absolute (95% CI)	
5	randomised trials	not serious	not serious	not serious	serious [b]	none	81	67	-	MD **0.41 lower nmol of DNPH/g/dl of proteins** (0.65 to 0.16 lower)	⨁⨁⨁◯ MODERATE
3	randomised trials	not serious	Not serious	not serious	serious [b]	none	41	27	-	MD **0.28 lower nmol of DNPH/g/dl of proteins** (0.61 lower to 0.05 higher)	⨁⨁⨁◯ MODERATE
3	randomised trials	not serious	Not serious	not serious	serious [b]	none	41	27	-	MD **0.98 lower nmol of DNPH/g/dl of proteins** (1.31 to 0.65 lower)	⨁⨁⨁◯ MODERATE
4	randomised trials	not serious	serious [a]	not serious	serious [b]	none	47	33	-	MD **1.02 lower nmol of DNPH/g/dl of proteins** (1.50 to 0.54 lower)	⨁⨁◯◯ LOW
2	randomised trials	not serious	not serious	not serious	serious [b]	none	29	15	-	MD **1.15 lower nmol of DNPH/g/dl of proteins** (1.79 to 0.52 lower)	⨁⨁⨁◯ MODERATE
1	randomised trials	not serious	serious [c]	not serious	serious [b,c]	none	21	7	-	Not pooled	⨁⨁◯◯ LOW

CI: Confidence interval; **MD:** Mean difference.

Explanations

a. Forest plots shows severe inconsistency

b. The total number of participants in this comparison is lower than optimal information size

c. Single study
